# The Toxin-Antitoxin Systems of the Opportunistic Pathogen *Stenotrophomonas maltophilia* of Environmental and Clinical Origin

**DOI:** 10.3390/toxins12100635

**Published:** 2020-10-01

**Authors:** Laurita Klimkaitė, Julija Armalytė, Jūratė Skerniškytė, Edita Sužiedėlienė

**Affiliations:** Institute of Biosciences, Life Sciences Center, Vilnius University, LT-1025 Vilnius, Lithuania; laurita.klimkaite@gmail.com (L.K.); jurate.skerniskyte@gmc.vu.lt (J.S.)

**Keywords:** *Stenotrophomonas maltophilia*, opportunistic pathogen, clinical origin, environmental origin, toxin-antitoxin system, biofilm, antibiotic resistance

## Abstract

*Stenotrophomonas maltophilia* is a ubiquitous environmental bacterium that has recently emerged as a multidrug-resistant opportunistic pathogen causing bloodstream, respiratory, and urinary tract infections. The connection between the commensal environmental *S. maltophilia* and the opportunistic pathogen strains is still under investigation. Bacterial toxin–antitoxin (TA) systems have been previously associated with pathogenic traits, such as biofilm formation and resistance to antibiotics, which are important in clinical settings. The same species of the bacterium can possess various sets of TAs, possibly influencing their overall stress response. While the TA systems of other important opportunistic pathogens have been researched, nothing is known about the TA systems of *S. maltophilia*. Here, we report the identification and characterization of *S. maltophilia* type II TA systems and their prevalence in the isolates of clinical and environmental origins. We found 49 putative TA systems by bioinformatic analysis in *S. maltophilia* genomes. Despite their even spread in sequenced *S. maltophilia* genomes, we observed that *relBE*, *hicAB*, and previously undescribed COG3832-ArsR operons were present solely in clinical *S. maltophilia* isolates collected in Lithuania, while *hipBA* was more frequent in the environmental ones. The kill-rescue experiments in *Escherichia coli* proved *higBA*, *hicAB*, and *relBE* systems to be functional TA modules. Together with different TA profiles, the clinical *S. maltophilia* isolates exhibited stronger biofilm formation, increased antibiotic, and serum resistance compared to environmental isolates. Such tendencies suggest that certain TA systems could be used as indicators of virulence traits.

## 1. Introduction

*Stenotrophomonas maltophilia* is a bacterium of class *Gammaproteobacteria*, ubiquitously found in the natural environment (soil, plants, animals, foods, or aqueous habitats) [1]. Recently, *S. maltophilia* came into attention as a globally emerging multidrug-resistant opportunistic pathogen causing nosocomial and community-acquired infections to immunocompromised patients [2]. The mortality rate of *S. maltophilia* infection can exceed 35% [3], with the World Health Organization (WHO) listing *S. maltophilia* as one of the leading multidrug-resistant pathogens [4]. The most known *S. maltophilia* features contributing to its successful adaptation in clinical environments include intrinsic antibiotic resistance mechanisms and its ability to form biofilms [5], although other less characterized factors (synthesis of extracellular enzymes, bacterial motility, and quorum sensing) are also known to influence its pathogenesis [6]. *S. maltophilia* is also known as a highly diverse species, since numerous studies have revealed genotypic diversity between clinical *S. maltophilia* isolates [7,8,9], even when analyzing samples from the same hospital [10,11]. Moreover, no significant genotypic or phenotypic differences between clinical and environment *S. maltophilia* isolates could be identified [12]. There is still an open question whether all the members of this species can become virulent and is there a specific marker to distinguish clinical and environmental *S. maltophilia* isolates.

The ability of pathogens to adapt to the clinical environment and to develop resistance to treatment can often be attributed to their ability to quickly exchange genetic information [13]. Gaining antibiotic resistance genes or virulence factors increases the chances of survival and gives rise to nosocomial opportunistic pathogens [14]. The ability of the bacterial species to mobilize their DNA could, therefore, be considered as a virulence factor in itself.

Toxin–antitoxin (TA) systems are abundant genetic elements mostly found in prokaryotes to encode a toxic protein, which inhibits cell growth and an antitoxin molecule (protein or RNA), which protects cells from toxin effects [15]. Human pathogenic bacteria have been shown to possess multiple sets of TAs, the type II TA systems, consisting of toxin and antitoxin proteins, being the most represented module and encoded by both plasmids and chromosomes [16]. Once the bacteria acquires DNA encoding the TA system, it becomes difficult to lose it due to the toxin’s higher stability and eventual suppression of the cell growth in case of loss of the genes [17]. Apart from being addictive, other functions have been proposed for the TA systems, such as a role in virulent phenotype and alleviating bacterial survival under stress conditions [18]. Moreover, the connection between TA systems and virulence traits, such as biofilm formation [19,20] and host colonization [18], was demonstrated. Interestingly, the same species can have various sets of TA systems [21], indicating their ability to be transferred horizontally. Analysis of the prevalence of TA systems in the genomes of 12 dangerous epidemic bacteria has shown that pathogens encoded a higher number of TA systems and reduced genomes compared to their closest non-pathogenic relatives [22]. This suggests that TAs might participate in the manifestation of the evolved pathogenic phenotype. The pathogenic *Mycobacterium tuberculosis* represents the prominent example, which genome encodes a remarkably high number of TA systems compared with nonpathogenic *Mycobacterium* strains [23]. Altogether, a possible TA systems role in bacteria pathogenicity is possible.

In this study, we aimed to identify and characterize previously undescribed *S. maltophilia* type II TA systems, as well as to evaluate and compare the abundance of these TA modules in clinical and environmental *S. maltophilia* isolates. Additionally, we accessed virulence-related features of *S. maltophilia,* including antibiotic and serum resistance, biofilm, pellicle, and capsule formation in order to find out if the correlation between the presence of TA systems and virulence-associated traits is apparent in *Stenotrophomonas*. We observed a difference in TA content and virulence-related characteristics between *S. maltophilia* isolates of clinical and environmental origins.

## 2. Results

### 2.1. Detection of S. maltophilia TA Systems in Genomes

**Figure 1:**
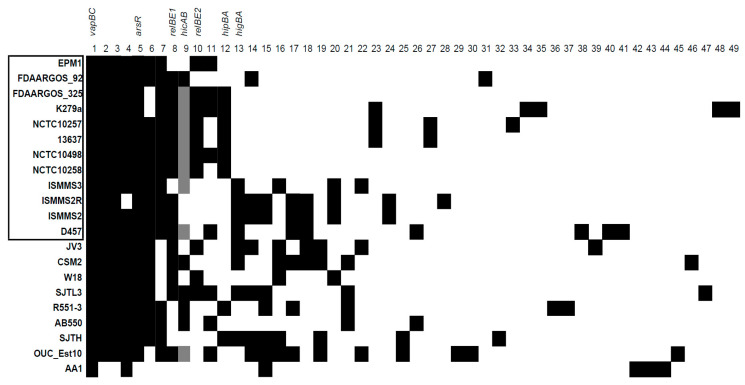
The predicted toxin–antitoxin (TA) systems in *S. maltophilia* genomes. The genomes with names bracketed in black rectangle were isolated from clinical sources. Black squares indicate the presence of a TA system, whereas grey squares indicate the presence of a pseudogene version. The information about the presented TA systems (1–49) is described in Table S1. TA systems analyzed in this work are indicated above.

To date, there are several tools available to detect TA systems in bacterial genomes: TADB 2.0 database tool TA-finder [24], RASTA-Bacteria [25], and TASmania [26]. However, only TA-finder was useful in our case, since RASTA-Bacteria limited the size of the analyzed DNA fragments, while TASmania relied on the Ensembl database, which at the time of this search did not have *S. maltophilia* genomes. On the date of our analysis, 21 fully assembled genomes were available to analyze with TA-finder. If the TA pair contained the same predicted domains, they were considered homologous; if they matched with a protein of a different domain, they were considered a distinct TA pair (e.g., RelE-Xre and RelE-RHH were considered as two distinct TAs). The presence of each predicted TA system was checked against all the genomes using BLAST. The final results for 49 detected TA systems are presented in Figure 1. Interestingly, some of the TA system genes were not identified in the genomes by TA-finder, but their presence was later detected by BLAST. After additional analysis, we found that the gene sequences in those genomes contained mutations that disrupted the reading frame, which could be due to sequencing errors or genuine mutations. The possible pseudogenes belonging to TAs are indicated in Figure 1.

**Figure 2:**
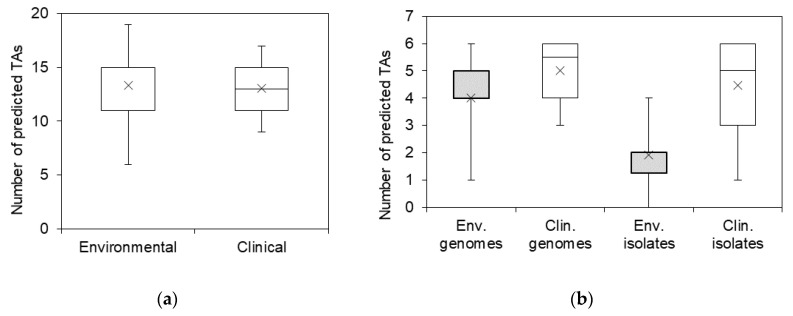
The prevalence of predicted TA systems in sequenced *S. maltophilia* genomes and *Stenotrophomonas* spp. isolates. (**a**) The distribution of total predicted TA systems in annotated *S. maltophilia* genomes; (**b**) the distribution of selected most prevalent TA systems in annotated genomes of *S. maltophilia* and in isolates of clinical and environmental origin. Boxes indicate upper and lower quartiles, whiskers indicate minimum and maximum values, and crosses indicate mean values.

As can be seen from Figure 1, there were no clear TA distribution patterns characteristic to annotated genomes of *S. maltophilia* from clinical or environmental origins. The average number of predicted TA systems per genome did not significantly differ between the *S. maltophilia* of both origins (Figure 2a).

Not all gene pairs predicted as TA systems are active TA modules [23,27,28]. Therefore, we aimed to check if the prevailing predicted TA systems of *S. maltophilia* were functional. For functional analysis, the TA systems that were present in at least 1/3 of the analyzed genomes were chosen. To increase the likelihood of the selected operons acting as TA systems, we chose TAs where both genes were no longer than 1 kb [25] (with the exception of *hipBA* system, where the toxin gene is known to be larger) [29]. The distance between the toxin and antitoxin genes was set to be 50 bp or less, which increased the possibility that the genes belonged to an operon and were expressed from a single transcript. The genes, which were clearly annotated for a function and not related to TAs (e.g., antibiotic resistance genes, membrane transporters), were excluded from further analysis. The final set of seven TAs, selected for functional characterization, is presented in Table 1.

The majority of the selected systems belonged to well-known type II TA families. There were three TA systems belonging to the *relBE* family: *relBE1* (containing RelE-Xre domains), *relBE2* (RelE-RHH domains), and *higBA* (HigB-Xre domains). However, one of the most common predicted TAs in *S. maltophilia*, containing COG3832-ArsR domains, was the least described. It was predicted as a TA system bioinformatically more than 10 years ago [30], yet only once was it analyzed thoroughly [23].

The predicted TA systems were conservative not only in *S. maltophilia* but in *Stenotrophomonas* spp. with high sequence conservativity (>70%) (Table 1). Therefore, we decided to test the presence and spread of these TAs in both *S. maltophilia* and *Stenotrophomonas* spp. isolates from our collection.

### 2.2. The Prevalence of TA Systems in Stenotrophomonas spp. of Clinical and Environmental Origin

**Figure 3:**
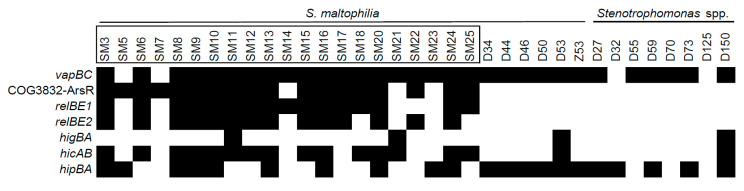
The prevalence of predicted TA systems in *Stenotrophomonas* spp. of clinical and environmental origins. The isolates with their names bracketed in black rectangles were derived from clinical sources. The presence of TAs, indicated as black boxes, was detected by PCR as described in the Materials and Methods section.

To detect selected TA systems, a set of primers was designed after aligning all available sequences for a TA system in a *Stenotrophomonas* genus and identifying the most conserved regions (Table S2). Next, the collection of *Stenotrophomonas* spp. of clinical (*n* = 21) and environmental (*n* = 14) origins (Figure 3) was screened for the presence of TAs. All isolates of clinical origin were attributed to *S. maltophilia* species, while six of the environmental isolates belonged to *S. maltophilia*. The rest of the environmental isolates were attributed to either *S. rhizophila* or *Stenotrophomonas* sp.

All of the examined TA systems were detected in at least four isolates (Figure 3). *vapC* and *hipBA* modules were the most prevalent (31 and 21 isolates, respectively), and were evenly spread in both clinical and environmental bacteria. However, *hipBA* was more common in isolates of environmental origin. Three TAs (*relBE1*, *relBE2*, and COG3832-ArsR) were prevalent in isolates of clinical origin yet were absent in environmental isolates. *hicAB* system was detected in the majority of clinical isolates and in only two isolates of environmental origin. Surprisingly, we observed a clear difference in TA profiles between *Stenotrophomonas* isolates of clinical and environmental origins, contradicting a similar distribution of these TAs in the sequenced genomes. We found that certain TAs were more likely to be present in the clinical isolates of *S. maltophilia*. Moreover, clinical isolates of *S. maltophilia* had more TA systems per isolate, compared to the environmental isolates (Figure 2b).

### 2.3. Functionality of S. maltophilia TA Systems

To test if the predicted TA systems were functional, we cloned toxin and antitoxin genes into separate inducible vectors to perform kill-rescue experiments in *E. coli*, as described in Materials and methods. The predicted toxin and antitoxin expression was induced with arabinose, and IPTG, respectively.

**Figure 4:**
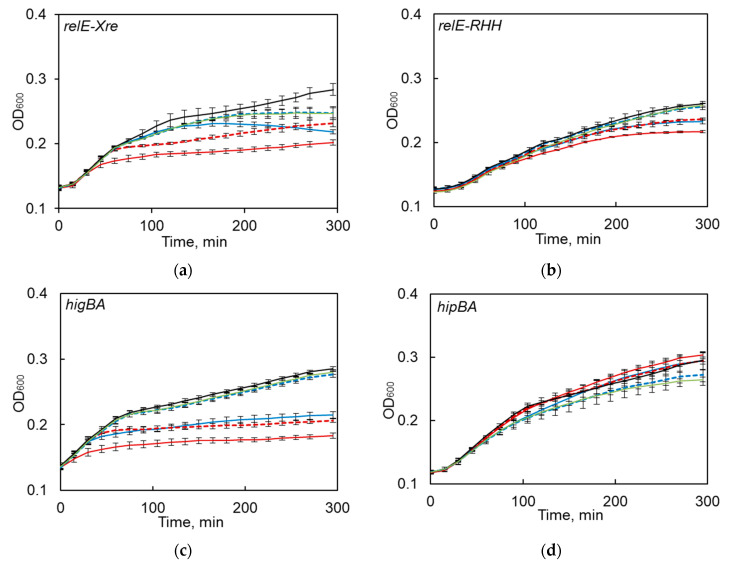

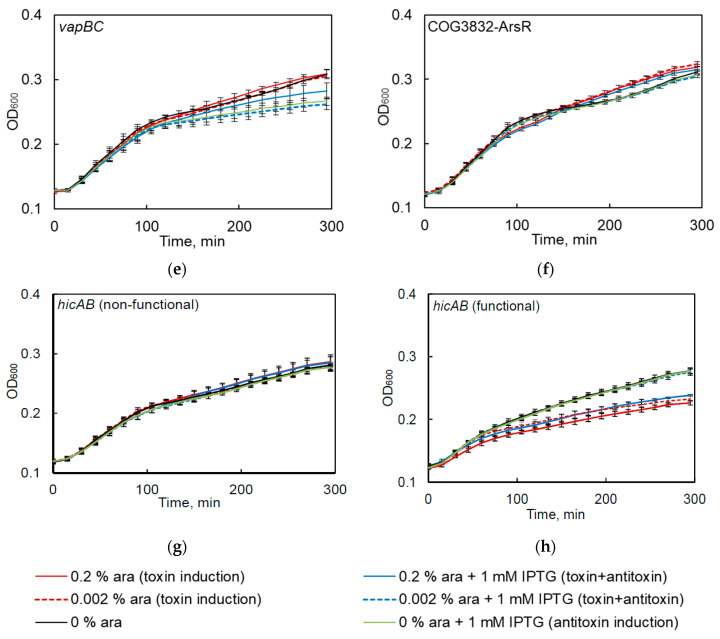
The functionality of predicted *S. maltophilia* TA systems as tested by the kill-rescue assay in *E. coli.* (**a**–**c**) the growth inhibition and restoration of functional TA systems; (**d**–**f**) the kill-rescue assays for the predicted TA that appear to be non-functional in *E. coli* system; (**g**,**h**) representative growth curves for a functional and a non-functional version of *hicAB*. The toxin and antitoxin genes were cloned into separate inducible plasmids, the toxin was induced by adding arabinose (ara), and its activity counteracted by inducing antitoxins with IPTG. The bacterial growth was measured as OD_600_, as described in the Materials and Methods section. The experiments were repeated at least three times; error bars indicate standard deviation.

Only *S. maltophilia relBE1*, *relBE2* and *higBA* TA systems, belonging to *relBE* family, were functional in kill-rescue experiments in *E. coli*, as evident from the growth inhibition upon induction of the toxin and growth restoration, when antitoxin was concomitantly induced (Figure 4a–c). The predicted toxins of *hipBA*, *vapBC*, *hicAB,* and COG3832-ArsR TA systems did not display any toxicity in the kill-rescue assay (Figure 4d–f).

We previously observed that one of the tested TAs (i.e., *hicAB*) was often present in the sequenced *S. maltophilia* genomes as a pseudogene (Figure 1). Therefore, when we first observed *hicAB* to be non-functional in the kill-rescue assay (Figure 4g), we decided to clone it from several other *S*. *maltophilia* isolates and to use the kill-rescue assay. Out of eight cloned versions of *hicAB*, three were non-functional, and five systems displayed a distinct kill-rescue phenotype. The growth of *E. coli* after the induction of a representative functional *S. maltophilia hicAB* is presented in Figure 4h. Sequencing of the cloned non-functional versions of *hicAB* revealed the same frameshift mutation in the toxin gene.

According to our results, *S. maltophilia* COG3832-ArsR, *hipBA,* and *vapBC* predicted TA systems were non-functional in *E. coli*. To find out, if this was due to the differences of the host or TAs, which were indeed non-functional, we created an expression system for *S. maltophilia* by using a broad-host range plasmid pBAD1 with an arabinose controlled promoter and cloned the predicted toxins. *S. maltophilia relE* toxin of the *relE-Xre* TA system was proven to be functional in *E. coli* and was thus used as a control. Further, *relE* toxin showed toxicity upon induction (not shown), while COG3832-ArsR, *hipBA*, and *vapC* toxins did not show any toxicity in *S. maltophilia* under the same induction conditions (not shown).

### 2.4. Characterization of Stenotrophomonas spp. Isolates

**Figure 5:**
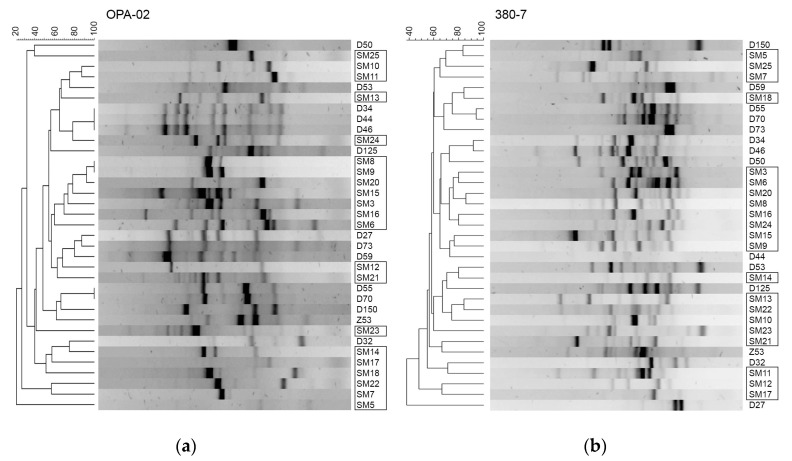
The genotyping of *Stenotrophomonas* spp. isolates. Random amplified polymorphic DNA (RAPD) assay was performed as described in the Materials and Methods section using OPA-02 (**a**) and 380-7 (**b**) primers (Table S2). The isolates bracketed in black rectangles were isolated from clinical sources.

Despite the similar distribution of predicted TAs observed in the *S. maltophilia* sequenced genomes of clinical and environmental origins (Figure 1), the most prevalent TA modules in bacterial isolates showed characteristic profiles regarding their origin, with clear prevalence for some TAs in clinical isolates (Figure 3). Since the majority of the clinical *S. maltophilia* isolates were obtained in the same hospital, we wanted to find out if there was a clonality between the analyzed isolates. The genotyping of *Stenotrophomonas* spp. isolates was undertaken by a random amplified polymorphic DNA (RAPD) assay with two sets of random primers as described in the Materials and Methods section. Each isolate significantly differed and was not grouped into clusters according to their origin (Figure 5). This thereby confirmed that the *S. maltophilia* of clinical origin were sporadic cases.

The genotyping of *Stenotrophomonas* spp. from clinical and environmental origins showed that isolates could not be grouped into clusters based on their origin of isolation. However, uneven distribution of TAs (i.e., the types of TAs and total TA number per isolate) among the isolates indicated that there were certain traits that distinguished the isolates of different origins. Therefore, we decided to compare other virulence-related traits of the isolates and see if the presence of TAs could be considered as a marker for virulence of the strain.

**Figure 6:**
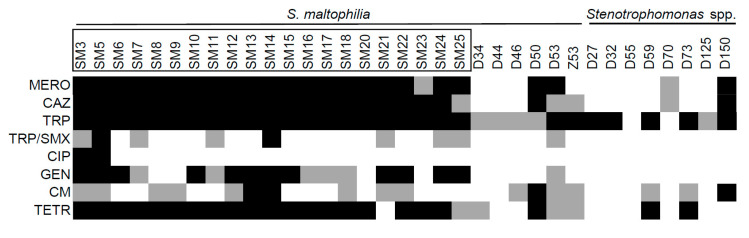
The resistance to antibiotics of *Stenotrophomonas* spp. of clinical and environmental origin. The isolates with names bracketed in black rectangles were isolated from clinical sources. Black squares indicate resistance; grey squares indicate intermediate resistance to antibiotic. The antibiotics are as indicated: meropenem (MERO), ceftazidime (CAZ), trimethoprime (TRP), trimethoprime-sulfametoxazole (TRP/SMX), ciprofloxacine (CIP), gentamicine (GEN), chloramphenicol (CM), and tetracyclin (TETR).

One of the characteristics that makes *S. maltophilia* a dangerous opportunistic pathogen is its innate resistance to antibiotics [31]. We tested the resistance of the isolates against a set of antibiotics (trimethoprime, chloramfenicol, gentamicine, ceftazidime, meropenem, and tetracyclin), including trimethoprime-sulfametoxazole and ciprofloxacine, which are commonly used against *S. maltophilia* infections. Resistance and sensitivity values were determined according EUCAST clinical breakpoints for *S. maltophilia* or *Pseudomonas aeruginosa*, if such data for *S. maltophilia* was not available. We observed significantly higher resistance profiles among the isolates of the clinical origin (Figure 6). Tetracyclin, ceftazidime, meropenem, and gentamicin were effective against most environmental isolates, while the majority of the clinical ones showed resistance.

**Figure 7:**
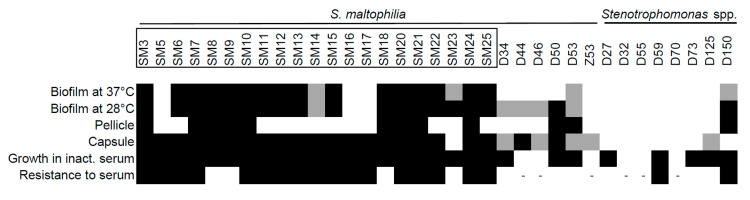
Biofilm formation and capsule-related characteristics of *Stenotrophomonas* spp. isolates. The biofilm, pellicle, capsule-forming phenotype, growth in inactivated serum and resistance to serum were determined as described in the Material and Methods section. The isolates with names bracketed in black rectangles were isolated from clinical sources. Black squares indicate the ability to form biofilm, pellicle, the presence of capsular polysaccharides (CPS), or the ability to grow in serum; grey squares indicate the ability to form weak biofilms or weak capsule-forming phenotype. “-” indicates that the isolates were not exposed to non-inactivated serum due to their inability to survive in inactivated serum.

The next virulence-related trait we assessed was biofilm formation, a complex community structure composed of adhesive bacteria and matrix components. Various bacteria can form biofilms on medical devices, such as catheters or intubation tubes, since it increases pathogen survival during stress (e.g., antibiotic pressure, desiccation, or laminar flow) [32]. Previous studies have shown the ability of *S. maltophilia* to form biofilms [33]. We first checked the ability of the isolates to form biofilms at 37 °C, as described in the Materials and Methods section, since this was an expected temperature inside the infected host. The majority of *S. maltophilia* from clinical origins formed strong biofilms (Figure 7, Figure S1). Only two environmental isolates displayed weak biofilm growth at 37 °C (Figure 7). However, the environmental isolates showed severely reduced growth at 37 °C, indicating that their inability to form biofilms could be due to suboptimal growth conditions. Therefore, we tested the ability of all the isolates to form biofilms at 28 °C, a temperature that resembled environmental (outside the host) conditions. No change in biofilm formation was observed for the clinical strains; however, several of the environmental strains increased their biofilm formation under permissive temperature. Thus, the results demonstrated a clear differentiation of the strains of different in ability to form biofilm at both 37 °C and 28 °C.

We then analyzed the ability of the isolates to form pellicle, a type of biofilm, which formed at the air–liquid interface. This specific structure is associated with the increased virulence of bacteria [34]. As can be seen in Figure 7, only the isolates with biofilm forming capacity were able to form pellicle, though this structure was not common to all such isolates and was more frequent in the clinical strains.

The ability to form capsule is considered as an important virulence trait in bacteria, together with resistance to antibiotics, biofilm formation, adhesiveness, desiccation, and other features [35,36,37]. The role of capsular exopolysaccharides in *S. maltophilia* virulence is still unknown. Therefore, we were interested to discover if bacterial isolates could produce polysaccharides that were indicative of capsule formation. The capsular polysaccharides were extracted and analyzed as described in the Materials and Methods section. The vast majority of clinical *S. maltophilia* isolates (95%) produced high molecular mass polysaccharides, which were associated with capsular polysaccharides (CPS). This phenotype was demonstrated by only a third (36%) of environmental *Stenotrophomonas* spp. isolates (Figure 7, Figure S2). However, *S. maltophilia* environmental isolates tended to produce capsule-associated polysaccharides more often than *Stenotrophomonas* of other species.

The ability to produce exopolysaccharide capsule is indicative of a strain’s ability to avoid killing by a host’s immune system [36,38]. We aimed to test if isolates differed in their ability to survive serum-mediated killing. First, we tested the ability of isolates to grow in a heat-treated serum with an inactivated complement system. All of the clinical isolates were able to grow, yet only half of the environmental isolates grew in the presence of inactivated serum (Figure 7). Next, we assessed the growth of *Stenotrophomonas* isolates in the presence of the active serum. The majority of clinical *S. maltophilia* strains were resistant to complement components when grown in an active serum, whereas only two of the environmental isolates showed resistance, thereby demonstrating an inability of environmental strains to resist a host immune system and cause an infection.

## 3. Discussion

In this study, we aimed to investigate the prevalence and profiles of TA systems in a newly emerging opportunistic pathogen, *S. maltophilia,* as well as to compare the TA presence with the manifestation of the virulence features of clinical and environmental isolates.

While the role of plasmid-borne TA systems is known as plasmid maintenance [39], the biological function of chromosome encoded TA systems is far less clear. It has been shown that chromosome encoded TA systems help stabilize genome integrity by preventing large scale genome reductions, which keeps genes needed for survival in unfavorable conditions [40,41] and causes growth arrest that helps bacteria survive environmental stress [39]. The most widespread type II TA system families affect the translation apparatus of a cell by cleaving or otherwise disrupting the functionality of mRNA (*relBE* (including *higBA*) [42,43], *hicAB* [44], and *mazEF* [45]) or tRNA (*vapBC* [46] and *hipBA* [29]). It has also been reported that TA systems function as virulence factors. Thus, a *higBA* TA system in *Pseudomonas aeruginosa* influences different virulence factor expression and biofilm formation [20]. *Mycobacterium tuberculosis MazEF* type TA systems play a role in pathogen intracellular survival inside the host [47]. A *Staphylococcus aureus* *SavRS* TA system functions as a regulator of virulence traits [48]. Moreover, previous studies have shown that the number of TA systems is enlarged in pathogenic strains compared with closely related nonpathogenic bacteria (*M. tuberculosis* encodes 88 putative TA systems [49], while non-harmful *M. smegmatis* encodes only three TA modules [50]). In this study, we investigated whether the same trend was characteristic to TA systems in the opportunistic pathogen *S. maltophilia*. The data observed on *vapBC* and *relBE* families, which are two major type II TA families [51,52], were in line with the observations on the prevalence of TA systems.

The amplicon detection-based screen of the TA systems in bacterial isolates revealed unexpected results, since *relBE1*, *relBE2,* and COG3832-ArsR TA systems were found solely in clinical *S. maltophilia* isolates and were not detected in environmental samples. Moreover, some preferences were observed for *hicAB* and *hipBA* TAs. The dissimilarity between observations of bioinformatic TA analysis in genomes available in the databases and our detection might be explained by the different geographic locations of bacterial sources. The bacteria analyzed in our study were isolated from soil in Lithuania, while the origin of *S. maltophilia* genomes in the databases varied greatly. However, the genotyping assay proved that the analyzed isolates did not belong to the same clonal group and showed high genetic diversity. Similar results were previously reported for *S. maltophilia* [53,54], supporting our findings about genotypic diversity among clinical and environmental *S. maltophilia* isolates from Lithuania.

In the present study, the putative COG3832-ArsR TA system was found in nearly all analyzed *S. maltophilia* genomes. COG3832-ArsR operon was previously proposed to be a TA system on the basis of comparative prokaryotic genomic analysis [30]. This module was found among 10 of the most induced TA systems in drug tolerant persister cells for *M. tuberculosis* [23], though functional analysis of this operon was not done in any TA system research. However, *S. maltophilia* COG3832-ArsR did not show toxicity in the kill-rescue assay and therefore its attribution to TA systems remains questionable. Altogether, functional analysis revealed that four of the seven analyzed *S. maltophilia* TA systems were functionally active TA operons in *E. coli*. The confirmed TAs belonged to previously described classes. To exclude the possibility of host incompatibility, we transferred the inactive toxins into *S. maltophilia*, yet no toxicity was observed. Interestingly, we identified two types of the same *S. maltophilia hicAB* TA systems via bioinformatic analysis in bacterial isolates. Previous studies have reported that TA system genes may contain mutations, which can result in nonfunctional pseudogene formation [55]. Loss of function mutations in virulence-associated genes can indicate genomic plasticity and adaptation to different environments [56], suggesting that *hicAB* TA systems may be favorable or, otherwise, unfavorable when living under particular conditions.

In our study, only the chromosome sequences of *S. maltophilia* were used for TA system prediction, however, the detected TA systems might be located on both chromosomes and mobile elements. TA systems are known to be transmitted via horizontal gene transfer [21,57]. It was shown that in *S. maltophilia,* trimethoprim/sulfamethoxazole resistance genes were found in class 1 integron and plasmid [58], indicating the ability *S. maltophilia* to acquire genetic information through horizontal gene transfer. Therefore, it is possible that some of the analyzed TA systems can spread via mobile elements, yet further studies are needed to confirm this possibility.

In this study, we demonstrated that clinical and environmental *Stenotrophomonas* isolates possessed not only distinct TA profiles but other important virulence-related traits. *S. maltophilia* is notorious for its multidrug resistance associated with intrinsic resistance factors, such as low membrane permeability, multidrug resistance efflux pumps, antibiotic-modifying enzymes, and ability to acquire resistance via mutations or acquisition of resistance genes through horizontal gene transfer [31]. Our study revealed a high antibiotic resistance of clinical *S. maltophilia* isolates, all of which displayed resistance to at least five out of eight antimicrobials. Environmental *Stenotrophomonas* isolates were more sensitive to antibiotics compared to bacteria of clinical origins, including meropenem (though *S. maltophilia* is known to have metallo-*β*-lactamases, providing resistance to various β-lactam antibiotics including carbapenems) [59,60]. The data on the antibiotic resistance level of clinical and environmental *S. maltophilia* isolates are contradictory [60,61,62], although it has been proposed that some mechanisms of antibiotic resistance occur in the environment and could remain in clinical isolates [63].

Biofilm formation is one of the best characterized *S. maltophilia* virulence traits. *S. maltophilia* forms biofilms on glass and different types of plastic [33], including medical devices (indwelling venous catheters [64], prosthetic valves [65], and lens implants [66]). Biofilm formation on biotic surfaces (e.g., human epithelial respiratory cells) has also been reported [67,68]. In this study, we showed that clinical and environmental *S. maltophilia* isolates differed in biofilm formation abilities, which was in line with previous studies [9,69]. Pellicle is a type of biofilm, formed at the air–liquid interface [70] and protecting bacterial population from environmental stresses. Further, it can increase survival rates by increasing accessibility to oxygen [71]. Less than a half of both clinical and environmental isolates were able to form pellicle, indicating that this phenotype may not be directly linked to virulence traits in *S. maltophilia*.

Although the ability of *S. maltophilia* to form capsule has not been described, studies of capsule functions in other bacterial pathogens showed its crucial role in the protection from unfavorable environmental conditions, including antibiotic treatment, host immune response, and resistance to disinfectants and heat [72,73,74,75]. Based on the separation of exopolysaccharides on SDS-PAGE gel, in some of the strains we observed the fraction of high molecular mass polysaccharides, as well as fractions resembling lipopolysaccharides [76]. Typically, such fractions represent capsular exopolysaccharides [37]. We demonstrated that CPS associated high molecular mass exopolysaccharides were detected in nearly all clinical isolates, while only 36% of environmental *Stenotrophomonas* spp. isolates displayed this phenotype. However, the majority of environmental isolates belonging to the *S. maltophilia* species did synthesize extracellular polysaccharides. Therefore, further studies are needed to investigate the importance of capsules in environmental isolates. Nevertheless, the presence of the capsule phenotypes among clinical *S. maltophilia* isolates supports the idea that capsule is beneficial to helping unfavorable conditions and host immune responses survive [38]. The hospital-related strains were also distinguished by increased resistance to serum-induced stress, especially when compared to strains of environmental origin. The ability of isolates to grow at 37 ºC (temperature of the host) and resist the complement system of the active serum indicates the increased infectivity potential of these strains. Some of the environmental isolates did not grow even in the heat-inactivated serum, which indicates the inability of these strains to multiply inside the host even before the encounter with the immune system. This could hint at the inability of environmental isolates to persist and grow due to improper nutrients and blood serum composition.

Based on the phenotypes of clinical and environmental *S. maltophilia* and genotyping results, we can assume that the environment could be the primary source of opportunistic *S. maltophilia* infections. However, further development of virulence potential might include an exchange of genetic information and switching on potential virulence genes.

## 4. Conclusions

Both clinical and environmental *S. maltophilia* genomes contain a variety of type II TA systems, the most common of which belong to *vapBC*, *relBE*, *hipBA*, *hicAB,* and putative COG3832-ArsR families. While bioinformatics assay did not show differences in TA distribution between clinical and environmental TAs, the screening of our collection of isolates did. *relBE* and COG3832-ArsR operons were present solely in clinical *S. maltophilia* isolates, while *hipBA* was more frequent in the environmental isolates. Together with different TA composition, the clinical *S. maltophilia* isolates exhibited clearly stronger biofilm formation, as well as increased antibiotic and serum resistance compared to environmental isolates.

## 5. Materials and Methods

### 5.1. Bioinformatic Assays

In total, 21 complete *S. maltophilia* genomes from NCBI database (date of accession 2018-10-13) (Table S3) were analyzed using the TADB 2.0 database tool TA-finder [24]. To identify TA systems in *S. maltophilia* genomes, a basic local alignment search tool (BLAST) [77] was used. The Clustal Omega tool [78] was used for sequence alignments.

### 5.2. The Bacteria Used in the Study and Growth Conditions

Plasmids and strains used in this study are listed in Table 2. Clinical and environmental *Stenotrophomonas* spp. isolates are listed in Table 3. *Stenotrophomonas* spp. and *E. coli* were grown in an LB medium, unless indicated otherwise. *E. coli* and clinical *S. maltophilia* isolates were grown at 37 °C. Environmental *S. maltophilia* and *Stenotrophomonas* spp. isolates were grown at 28 °C, unless indicated otherwise. DNA was transformed to *E. coli* by heat shock [79] and electroporation was used for *S. maltophilia* [80]. Antibiotics (ampicillin 100 mg/L, chloramphenicol 30 mg/L, and kanamycin 60 mg/L) were used to maintain plasmids when needed.

### 5.3. Detection of TA Systems in Stenotrophomonas

For the detection of *S. maltophilia* TA systems, primers targeting conservative gene fragments were designed (Table S2) and PCR was performed using DreamTaq polymerase (Thermo Fisher Scientific).

### 5.4. Cloning of TA System Genes

All enzymes used for cloning were from Thermo Fisher Scientific and were used according to the manufacturer’s recommendations. For gene cloning, a high-fidelity Phusion polymerase was used with the primers listed in Table S2. A *hicAB* TA system was cloned using SM6, SM9, SM11, SM12, SM13, SM16, SM20, and SM24 isolate DNA as the template. For *relBE2* and *higBA* the isolate SM11 was used, for *relBE1, hipBA, vapBC,* and COG3832-ArsR the isolate SM9 DNA was used as a template. For TA functional analysis in *E. coli*, all toxin genes were cloned into pBAD30 plasmid; antitoxin genes were cloned into pUHcat plasmid. Both vectors were hydrolyzed with SphI, then overhangs were blunted with T4 polymerase. Final restriction was performed with HindIII. To clone the toxins into a broad host range pBAD1 vector, pBAD1_GFP plasmid was used. First, the pBAD1_GFP vector was hydrolyzed with XbaI and other steps were performed as described above.

### 5.5. Kill-Rescue Assay

The kill-rescue assay was performed as described previously [86]. Briefly, *E. coli* BW25113 F‘ strains harboring plasmids pBAD_tox and pUHEcat_antitox were grown overnight, diluted 1:500 into fresh medium supplemented with 0.2% glucose, and grown to its early exponential phase (optical density at 600 nm (OD_600_) = 0.12). The expression of toxin and/or antitoxin was then induced with arabinose (0.002% and 0.2%) and/or IPTG (1 mM), respectively. Growth was measured as OD_600_ in a Tecan Infinite M200 Pro plate reader at 37 °C with shaking.

### 5.6. Random Amplified Polymorphic DNA (RAPD) Assay

For the RAPD assay, DNA amplification was performed at the same time on the same thermocycler in a final reaction volume of 25 µL, containing 2.5 µL of 10× polymerase reaction buffer with KCl: 0.5 µL 10 mM dNTP, 1.25 µL 50 mM MgCl_2_, 2 µL 10 pmol/µL OPA-02 or 380-7 primer, 0.15 µL DreamTaq polymerase, and 5 µL of bacteria lysate.

Cycling conditions are as follows: initial denaturation at 95 °C for 2 min followed by 39 cycles of denaturation at 95 °C for 30 s, annealing at 36 °C/34 °C for 1 min (OPA-02 and 380-7 primers, respectively), extension at 72 °C for 2 min and final extension at 72 °C for 5 min.

PCR products were loaded on a 1% (*w/v*) agarose gel with 0.5 mg/mL of ethidium bromide (1 × TAE buffer, 7 V/cm voltage, 1.5 h). Electrophoresis gels were visualized with Bio Rad Molecular Imager and analyzed using GelCompar II software (Applied Maths) with the Dice coefficient set at 1% and band tolerance set at 1.5% using the UPGMA method.

### 5.7. The Evaluation of Antibiotic Resistance

Antimicrobial resistance analysis was performed by growing *Stenotrophomonas* isolates on agarose LB plates with selected concentrations of antimicrobial agents for 16 h at 28 °C. Control growth was considered as growth of isolates on the LB plates without antimicrobial agents. The isolate was considered as resistant to tested antibiotics only if growth visually looked the same as the control. In the case of significantly weaker growth, the isolate was referred to as an intermediate resistant and in the absence of growth, the isolate was considered as sensitive.

### 5.8. Biofilm Formation Assay

Biofilm formation experiments were performed as described elsewhere [38]. Briefly, overnight *Stenotrophomonas* spp. cultures (grown in TSB medium (Oxoid) at 37 ◦C for 16 h) were inoculated into the 150 μL TSB medium containing wells of 96 U-form polystyrene plate (30-fold dilution), then incubated stationary at 28  °C or 37  °C for 24 h. The OD_600_ of planktonic culture was measured and wells were washed 3 times with PBS buffer. Adherent cells were stained with 0.1% crystal violet dye for 15 min, then washed 5 times with the PBS buffer. Dye was dissolved in 96% ethanol for OD_580_ measurements. The OD_580_/OD_600_ ratio was calculated to normalize the number of biofilm forming cells to the total cell number.

### 5.9. Pellicle Formation Assay

The pellicle formation was performed as previously described [87]. Briefly, overnight cultures *Stenotrophomonas* spp. (grown 16 h in TSB medium at 37  °C) were inoculated into the TSB medium containing wells of a flat-bottom 12 well polystyrene microplate in a total volume of 3 mL (1000-fold dilution). The cultures were incubated stationary at 28  °C for 30  h. Results were evaluated qualitatively by monitoring the presence/absence of bacterial biomass formed in the air–liquid contact.

### 5.10. Extraction of Capsular Polysaccharides

The bacteria were grown on LB agar plates for 16 h and suspended in the PBS buffer. Capsular polysaccharide extraction and analysis were performed as described earlier [38]. Briefly, polysaccharides were released by vortexing for 30 s. After centrifugation at 9000× *g* for 10 min, polysaccharides were precipitated in 75% ice-cold ethanol. The pellet was resuspended in SDS sample buffer and boiled for 5 min. Samples were loaded on the 12% SDS-PAGE gels. After electrophoresis, gels were stained overnight with 0.1% (*w*/*v*) Alcian Blue.

### 5.11. Serum Resistance

Serum resistance was evaluated by measuring bacterial growth in LB medium, heat inactivated FBS (fetal bovine serum (Gibco, 12657029)), and active FBS. FBS was inactivated by incubation at 56 °C for 30 min with constant shaking. Overnight cultures were inoculated at ×1000 dilution to LB medium and 80% FBS (20% of LB medium) or inactive 80% FBS. Growth curves were measured at 37 °C with periodic shaking every 20 min using a Tecan Infinite M200 Pro microplate reader.

## Figures and Tables

**Table 1:** The characteristics of TA systems prevalent in annotated genomes of *S. maltophilia.*

Predicted TA Family	Protein Domains (T-A) ^1^	Found in Genomes (*n* = 21)	Conservativity of Operon (%) ^2^	Prevalence in Other Bacteria ^3^	Remarks
*vapBC*	PIN-AbrB	21	84	*Stenotrophomonas* spp., *Xantomonas* spp.	
-	COG3832- ArsR	20	90	*Stenotrophomonas* spp. and other genera	
*relBE*	RelE-Xre	15	73	*Stenotrophomonas* spp.	T before A
*relBE*	RelE-RHH	10	92	*Stenotrophomonas* spp.	
*relBE* *(higBA)*	HigB-Xre	8	78	*Stenotrophomonas* spp., *Pseudomonas* spp.	T before A
*hicAB*	HicA-HicB	14	79	*Stenotrophomonas* spp.	T before A
*hipBA*	HipA-Xre	8	70	*Stenotrophomonas* spp. (T common to *Burkholderia* spp.)	T is 440 a.a.

^1^ Predicted domains (domain-like); ^2^ in *Stenotrophomonas* spp.; ^3^ the prevalence of selected TA systems was identified by BLAST analysis against domain *Bacteria*; T: toxin, A: antitoxin.

**Table 2:** Strains and plasmids used in this study.

Strain/Plasmid	Description	Reference
*E. coli* JM107	*endA1 glnV44 thi-1 relA1 gyrA96 Δ(lac-proAB) [F’ traD36 proAB^+^ lacI^q^ lacZΔM15]* *hsdR17(R_K_^−^ m_K_^+^) λ^−^*	[81]
*E. coli* BW25113 F’	*proA^+^B^+^lacIqΔlacZ)M15 zzf::mini-Tn10* (KanR)	[82]
pBAD30	Expression plasmid	[83]
pUHE 25-2(cat)	Expression plasmid	[82]
pBAD1_gfp	*gfp* gene cloned into pBAD-MCS-1 vector	[84]

**Table 3:** Clinical and environmental *Stenotrophomonas* spp. isolates used in this study.

Isolate	Genus/Species	Origin	Source ^1^	Isolation Year
D27	*S. maltophilia/rhizophila*	Environmental	Soil (Conventional wheat)	2016
D32	*Stenotrophomonas* sp.	Environmental	Soil (Conventional wheat)	2016
D34	*S. maltophilia*	Environmental	Soil (Conventional wheat)	2016
D44	*S. maltophilia*	Environmental	Soil (Conventional wheat)	2016
D46	*S. maltophilia*	Environmental	Soil (Conventional wheat)	2016
D50	*S. maltophilia*	Environmental	Soil (Conventional wheat)	2016
D53	*S. maltophilia*	Environmental	Soil (Conventional wheat)	2016
D55	*Stenotrophomonas* sp.	Environmental	Soil (Organic rapeseed)	2016
D59	*Stenotrophomonas* sp.	Environmental	Soil (Organic rapeseed)	2016
D70	*S. rhizophila*	Environmental	Soil (Organic rapeseed)	2016
D73	*Stenotrophomonas* sp.	Environmental	Soil (Organic rapeseed)	2016
D125	*Stenotrophomonas* sp.	Environmental	Soil (Organic maize)	2016
D150	*Stenotrophomonas* sp.	Environmental	Soil (Conventional maize)	2016
Z53	*S. maltophilia*	Environmental	Fish (Carp)	2016
SM3	*S. maltophilia*	Clinical	NCI	2017
SM5	*S. maltophilia*	Clinical	VCCH LM	2018
SM6	*S. maltophilia*	Clinical	VCCH LM	2018
SM7	*S. maltophilia*	Clinical	VCCH LM	2018
SM8	*S. maltophilia*	Clinical	NCI	2019
SM9	*S. maltophilia*	Clinical	NCI	2019
SM10	*S. maltophilia*	Clinical	VCCH LM	2019
SM11	*S. maltophilia*	Clinical	VUH SK PD	2019
SM12	*S. maltophilia*	Clinical	VUH SK PD	2019
SM13	*S. maltophilia*	Clinical	VUH SK PD	2019
SM14	*S. maltophilia*	Clinical	VCCH LM	2019
SM15	*S.maltophilia*	Clinical	KCPHC	2019
SM16	*S. maltophilia*	Clinical	VCCH LM	2019
SM17	*S. maltophilia*	Clinical	NCI	2019
SM18	*S. maltophilia*	Clinical	NCI	2019
SM20	*S. maltophilia*	Clinical	NCI	2019
SM21	*S. maltophilia*	Clinical	NCI	2019
SM22	*S. maltophilia*	Clinical	NCI	2019
SM23	*S. maltophilia*	Clinical	NCI	2019
SM24	*S. maltophilia*	Clinical	NCI	2019
SM25	*S. maltophilia*	Clinical	NCI	2019

^1^ NCI—National Cancer Institute (Lithuania); VCCH LM—Vilnius City Clinical Hospital, Laboratory of Microbiology; VUH SK PD—Vilnius University Hospital Santaros Klinikos, Pediatrics Department, Division of Infectious Diseases; KCPHC—Kaunas City Public Health Center. The isolates from soil were isolated and described previously [85].

## Supplementary Materials

The following are available online at https://www.mdpi.com/2072-6651/12/10/635/s1, Figure S1: Biofilm formation of clinical and environmental *Stenotrophomonas* spp. isolates, Figure S2: The polysaccharide production of *Stenotrophomonas* spp. isolates of clinical and environmental origin, Table S1: TA systems detected by TA-finder in annotated *S. maltophilia* genomes, Table S2: Primers used in this study, Table S3: *S. maltophilia* genomes used in this study.
