# Supplementary material for: The Toxin-Antitoxin Systems of the Opportunistic Pathogen Stenotrophomonas maltophilia of Environmental and Clinical Origin
Source: Toxins (Basel). 2020 Oct 1;12(10):635. doi: 10.3390/toxins12100635 (PMC7650669; doi:10.3390/toxins12100635)
Supplement: Supplementary file 1 [file toxins-12-00635-s001.zip › toxins-902548 supplementary proof done/toxins-902548 supplementary proof.docx]

Supplementary Materials: The Toxin-Antitoxin Systems of the Opportunistic Pathogen Stenotrophomonas maltophilia of Environmental and Clinical Origin

Laurita Klimkaitė, Julija Armalytė, Jūratė Skerniškytė and Edita Sužiedėlienė


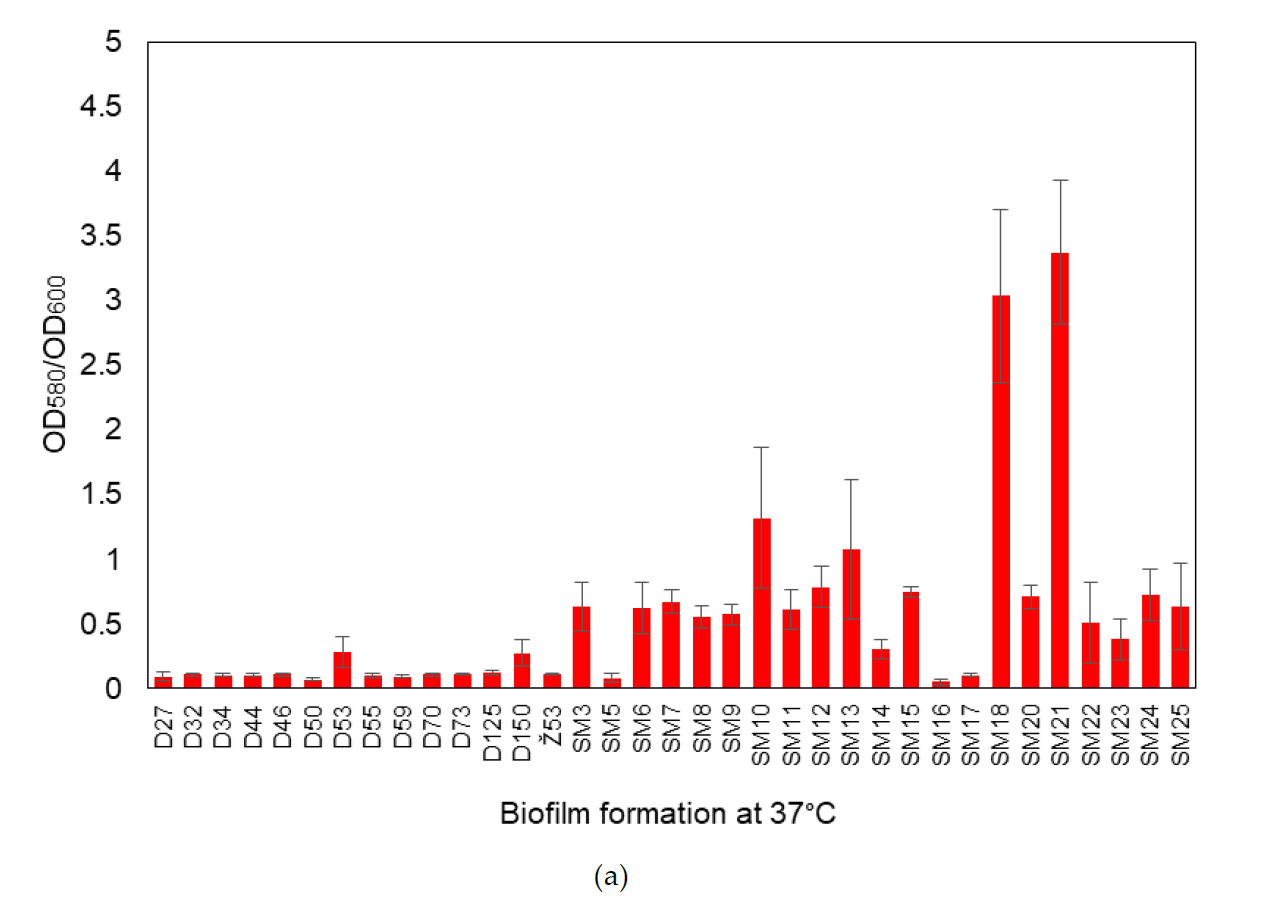


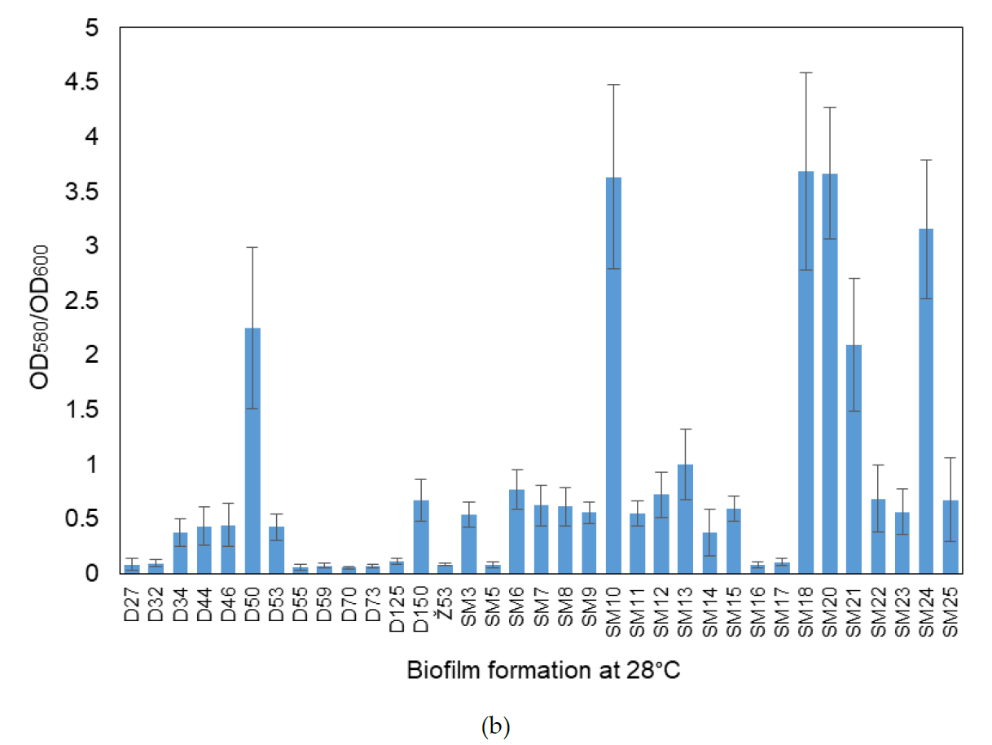


**Figure S1.** Biofilm formation of clinical and environmental *Stenotrophomonas* spp. isolates. The biofilms were grown as described in Materials and methods, and measured after staining of adherent cells with crystal violet dye. The measurements were normalized by calculating OD_580_ / OD_600_ ratio.


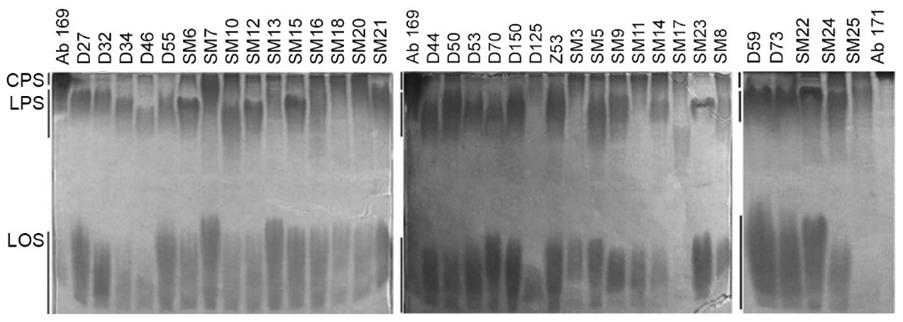


**Figure S2.** The polysaccharide production of *Stenotrophomonas* spp. isolates of clinical and environmental origin. The isolated fractions were separated by SDS-PAGE and stained with Alcian Blue as described in Materials and methods. Lines on the left side of the gels indicate the positions of capsular polysaccharides (CPS), lipopolysaccharides (LPS) and lipooligosaccharides (LOS). Ab 169 and Ab 171 are previously described *Acinetobacter baumannii* isolates, that have been shown to have capsule [37].

**Table S2.** Primers used in this study.

| **Primer** | **Primer sequence (5’-> 3’)** | **Primer function** | **Reference** |
| --- | --- | --- | --- |
| Steno_vapBC_det_F | GGRCAGATCACCYTGCCCAA | Putative *vapBC* TA system detection | This work |
| Steno_vapBC_det_R | CACGATCAGCTTCARGCCCTTG |  |  |
| Steno_hicAB_det_F | GAAGCTYGCDGGRCTGACGTA | Putative *hicAB* TA system detection | This work |
| Steno_hicAB_det_R | GCAGGCTGATRTTGACCCGCT |  |  |
| Steno_relE-Xre_det_F | ATCATGBCACGYGTYTTCCGCAC | Putative *relE-Xre* TA system detection | This work |
| Steno_relE-Xre_det_R | GGYTTCTTBTCGCCCTGYTCCCA |  |  |
| Steno_32_ArsR-1_det_F | GACCCSACCCGDTGCGCGAT | Putative  COG3832-ArsR  TA system detection | This work |
| Steno_32_ArsR-1_det_R | GGCACCACCACTGGCGCAG |  |  |
| Steno_relE-RHH_det_F | CCACCATGAACATTTCGCTGAC | Putative *relE-RHH* TA system detection | This work |
| Steno_relE-RHH_det_R | CGACGAACCAATATCGGGGTTG |  |  |
| Steno_higBA_det_F | CTCTATGAGCGGGGCGACAT | Putative *higBA* TA system detection | This work |
| Steno_higBA_det_R | GCCTGCATCGCCAGCCAC |  |  |
| Steno_hipBA_det_F | GCGACCCKGCATCGGCAAG | Putative *hipBA* TA system detection | This work |
| Steno_hipBA_det_R | TCCTGCGCACCGGCGATGG |  |  |
| vapBC_pUH_antitox_F | CATGGAAGCCACCGTTGCAGAACG | Cloning of putative *vapBC* TA system antitoxin into pUHEcat | This work |
| vapBC_pUH_antitox_R | TAATAAGCTTGGTGAATCGACGGCGATCAT |  |  |
| vapBC_pBAD_tox_F | GATGATCGCCGTCGATTCACCGG | Cloning of putative *vapBC* TA system toxin into pBAD plasmids | This work |
| vapBC_pBAD_tox_R | TATTAAGCTTTCAGGCTTGCGGCACGATCAG |  |  |
| ArsR-32_pUH_Antitox_F | TATGGGTAAGTATGATCCCGCCAT | Cloning of putatative *ArsR-COG3832*  TA system antitoxin to pUHEcat | This work |
| ArsR-32_pUH_Antitox_R | TAATAAGCTTCGGCTGATGACCAGATCGGT |  |  |
| ArsR-32_pBAD_tox_F | CATGGCCGTAGACGCAGGTAACAA | Cloning of putative COG3832-ArsR TA system toxin into pBAD plasmids | This work |
| ArsR-32_pBAD_tox_R | TAATAAGCTTTCAGCCGTCGAAGAAGCCCA |  |  |
| hipBA_antitox_F | TATGGACAGGCCGCTGCACAC | Cloning of putatative *hipBA* TA system antitoxin into pUHEcat | This work |
| hipBA_antitox_R | TAATAAGCTTTCACCACTCCAATGACGACGT |  |  |
| hipBA_tox_F | CATGGGTACGCTGCAGGTCTGGA | Cloning of putative *hipBA* TA system toxin into pBAD plasmids | This work |
| hipBA_tox_R | TATTAAGCTTTCAAGCCTCGGGGGCCATGCTG |  |  |
| hicAB_antitox_F | CATGCGATATCCAGTCTTGATCGA | Cloning of putatative *hicAB* TA system antitoxin into pUHEcat | This work |
| hicAB_antitox_R | TAATAAGCTTTCAGCCCAATGCCGCGAGAT |  |  |
| hicAB_tox_F | CATGAAGAGCAGGGACCTCATCCG | Cloning of putative *hicAB* TA system toxin into pBAD plasmids | This work |
| hicAB_tox_R | TATTAAGCTTCTACGTCAGTCCTGCGAGCTTC |  |  |
| relE-RHH_antitox_F | CATGGCCACCATGAACATTTCG | Cloning of putatative *relE-RHH* TA system antitoxin to pUHEcat | This work |
| relE-RHH_antitox_R | TATTAAGCTTTCACTTGCGGTTCCGTGCA |  |  |
| relE-RHH_tox_F | AATGAAGCCCTCGCACTGGTCC | Cloning of putative *relE-RHH* TA system toxin to pBAD vectors | This work |
| relE-RHH_tox_R | TAATAAGCTTTCAGTATCCTGGATGCGACGAAC |  |  |
| relE-Xre_tox_F | TATGATCATGTCACGTGTCTTCCG | Cloning of putative *relE-Xre* TA system toxin to pBAD vectors | This work |
| relE-Xre_tox_R | TATTAAGCTTTCARSSGTCTTYGTCATGGCHRATCTC |  |  |
| relE-Xre_antitox_F | CATGACAAAGACCCTTGA | Cloning of putatative *relE-Xre* TA system antitoxin to pUHEcat | This work |
| relE-Xre_antitox_R | TATTAAGCTTTTACAGGACTGCTTCGAG |  |  |
| higBA_tox_F | CATGATCGTCAGCTTCAGGCA | Cloning of putative *higBA* TA system toxin into pBAD plasmids | This work |
| higBA_tox_R | TATTAAGCTTCTAGTGGTAATCAAGGTAATC |  |  |
| higBA_antitox_F | GATGCCGTTGCATGATCCGCC | Cloning of putatative *higBA* TA system antitoxin into pUHEcat | This work |
| higBA_antitox_R | TATTAAGCTTCTACCGCTCTATCCTCACTGCATCCT |  |  |
| OPA-02 | TGCCGAGCTG | RAPD analysis | [83] |
| 380-7 | GGCAAGCGGG | RAPD analysis | [84] |

**Table S3.** *S. maltophilia* genomes used in this study.

| Genome | Accession | Source |
| --- | --- | --- |
| *Stenotrophomonas maltophilia* K279a | AM743169.1 | Clinical |
| *Stenotrophomonas maltophilia* R551-3 | CP001111.1 | Environmental |
| *Stenotrophomonas maltophilia* JV3 | CP002986.1 | Environmental |
| *Stenotrophomonas maltophilia* D457 | HE798556.1 | Clinical |
| *Stenotrophomonas maltophilia* ISMMS3 | CP011010.1 | Clinical |
| *Stenotrophomonas maltophilia* ISMMS2 | CP011305.1 | Clinical |
| *Stenotrophomonas maltophilia* ISMMS2R | CP011306.1 | Clinical |
| *Stenotrophomonas maltophilia* AA1 | CP018756.1 | Environmental |
| *Stenotrophomonas maltophilia* OUC_Est10 | CP015612.1 | Environmental |
| *Stenotrophomonas maltophilia* AB550 | CP028899.1 | Environmental |
| *Stenotrophomonas maltophilia* FDAARGOS_325 | CP022053.2 | Clinical |
| *Stenotrophomonas maltophilia* CSM2 | CP025298.1 | Environmental |
| *Stenotrophomonas maltophilia*FDAARGOS_92 | CP014014.1 | Clinical |
| *Stenotrophomonas maltophilia* SJTH1 | CP027562.1 | Environmental |
| *Stenotrophomonas maltophilia* W18 | CP028358.1 | Environmental |
| *Stenotrophomonas maltophilia* SJTL3 | CP029773.1 | Environmental |
| *Stenotrophomonas maltophilia* NCTC10257 | LT906480.1 | Clinical |
| *Stenotrophomonas maltophilia* NCTC10258 | LS483377.1 | Clinical |
| *Stenotrophomonas maltophilia* NCTC10498 | LS483406.1 | Clinical |
| *Stenotrophomonas maltophilia* 13637 | CP008838.1 | Clinical |
| *Stenotrophomonas maltophilia* EPM1 | CM001824.1 | Clinical |
